# Employment status and health: understanding the health of the economically inactive population in Scotland

**DOI:** 10.1186/1471-2458-12-327

**Published:** 2012-07-12

**Authors:** Judith Brown, Evangelia Demou, Madeleine Ann Tristram, Harper Gilmour, Kaveh A Sanati, Ewan B Macdonald

**Affiliations:** 1 Healthy Working Lives Group, Institute of Health and Wellbeing, College of Medical, Veterinary and Life Sciences, 1 Lilybank Gardens, University of Glasgow, Glasgow, G12 8RZ, UK; 2 Medical Statistics, School of Mathematics and Statistics, College of Science and Engineering, University of Glasgow, Glasgow, G12 8QW, UK

## Abstract

**Background:**

Although the association between health and unemployment has been well examined, less attention has been paid to the health of the economically inactive (EI) population. Scotland has one of the worst health records compared to any Western European country and the EI population account for 23% of the working age population. The aim of this study is to investigate and compare the health outcomes and behaviours of the employed, unemployed and the EI populations (further subdivided into the permanently sick, looking after home and family [LAHF] and others) in Scotland.

**Methods:**

Using data from the 2003 Scottish Health Survey, the differences in health and health behaviours among the employed, unemployed and the subgroups of the EI population were examined.

**Results:**

Both low educational attainment and residence in a deprived community were more likely in the permanently sick group. The LAHF and the unemployed showed worse self-reported health and limiting longstanding illness compared to the employed but no significant differences were observed between these groups. The permanently sick group had significantly poorer health outcomes than all the other economic groups. Similar to the unemployed and LAHF they are more likely to smoke than the employed but less likely (along with LAHF and ‘others’) to exhibit heavy alcohol consumption. Interestingly, the LAHF showed better mental health than the rest of the EI group, but a similar mental health status to the unemployed. On the physical health element of lung function, the LAHF were no worse than the employed.

**Conclusion:**

While on-going health promotion and vocational rehabilitation efforts need to be directed towards all, our data suggests that the EI group is at higher risk and policies and strategies directed at this group may need particular attention.

## Background

A plethora of research has confirmed the link between poor health outcomes and unemployment [1-7] and a recent study found this relationship was consistent across 23 European countries [8]. The literature highlights that the health effects of unemployment could be induced by socio-economic factors, such as financial strain and poverty [1,3,4]. In addition, individual risk factors, such as lack of exercise, alcohol abuse and smoking, can substantially contribute to the increased relative mortality risk associated with unemployment. [1,2,5,9,10]. However, debate continues over whether unemployment causes deterioration of health or whether those at higher risk of unemployment were in poorer health prior to becoming unemployed [7,11].

The evidence review of Waddell and Burton (2006) has confirmed that being in work is generally better for health and wellbeing than being out of work [7]. Evidence suggests that work has beneficial long-term effects and generally the majority of people in healthy and safe work live longer than those out of work [7,12]. Moving from work to a state of unemployment increases mortality risk [7], and experiencing job loss even once, increases the risk of premature mortality [13]. Contrarily, moving from being unemployed to being employed, is reported to lead to increased self-esteem, improved general and mental health and reduced psychological distress [7,14,15].

In the context of employment status, it is common to describe individuals as employed versus unemployed [16]. However, when considering how the working age population (WAP) is involved with the labour market a third group emerges - the economically inactive (EI) [16]. More recently the unemployed and EI have been collectively referred to as the ‘workless’ population [17]. The EI include those people who are not in work and who do not satisfy all the criteria for the International Labour Organisation’s (ILO) unemployment definition [16], and are therefore neither working nor actively seeking work [16]. The EI population is a rather heterogeneous group made up of three main groups - the permanently sick, many of which will be receiving sickness-related benefits (in Scotland and the UK this would be incapacity benefit or the new Employment and Support Allowance (ESA) [18]), those that by choice or necessity are carers and are looking after home and family (LAHF), and others such as students and retired individuals. Therefore, the health status characteristics of this group are expected to cover a wide spectrum.

Currently, the number of people classified as being EI in Scotland is 781,200, which is equivalent to 23% of the WAP [19]. Scotland has one of the worst health records amongst those of working age compared to any Western European country [20], and sickness and disability are generally associated with being EI [21]. While a number of studies have investigated the health outcomes associated with employment and unemployment [1-7,9,22-24], the information available on health outcomes associated with being EI is still sparse [21,22,25-29], while even fewer studies have looked at the subcategories within the EI population [27-30]. A Scottish longitudinal study found that few of those EI due to sickness or disability in 1991 had moved into employment in 2001. However, for those that did move into employment, their risk of reporting a limiting longstanding illness (LLI) was decreased [21].

Ritchie et al. (2005) investigated the geographic distributions of the unemployed and EI in the UK, but not the health status of these particular groups [17]. It was revealed that, overall, workless individuals lived in more deprived areas [17,21]. Fone et al. (2006) examined the associations between mental health problems and economic inactivity at ward level using the Welsh Health Survey [25]. Their study showed mental health was significantly associated with the ward deprivation score and the effect was strongest for the EI [25]. Honkonen et al. (2007) concluded that the EI were similar to the unemployed in exhibiting a higher relative risk of having a mental health problem compared to the employed [26], but did not consider the composition of the EI. A similar situation is observed for LLI, where unemployment and economic inactivity are high predictors of the on-set of LLI [22]. In a study conducted in England and Wales, both mortality and LLI were lowest amongst those in work, much higher for the permanently sick, while all others (investigated as a single group minus the retired population), exhibited rates that fell between those for the employed and permanently sick [31].

Dissecting the EI group into its subcategories gives more insight into the characteristics of the people within these, the health issues, and needs associated with each subgroup. In an earlier study, Thomas et al. (2005) used the British Household Panel Survey to investigate the health impact of transitions between employment and states of worklessness and demonstrated that moving from work to worklessness was associated with psychological distress. The effect was most pronounced for moving from formal employment to long-term illness, while moving into the family-care or maternity-leave category was not significantly different to moving to unemployment, retirement or full time study [29]. Similarly, using the 2001 English Census, Bambra and Popham (2010), showed that when breaking down the EI group, the permanently sick were significantly different in terms of general health than all groups, whereas the LAHF and others were not that different from the unemployed [27].

While much of the research in employment status and health has been concerned with the employed and unemployed groups, less research has been undertaken on the health of the EI population, and specifically on the subcategories of this group. This is especially important in the current climate where welfare reform is making it mandatory for individuals from all categories of the workless population to engage, possibly for the first time, or re-engage after a period of absence, with the labour market [32-34]. The few studies that have been published are often limited by not exploring health outcomes at all or simply focusing on one health issue and not examining the breakdown of the EI population. Additionally, there is a lack of regional and up-to-date information for Scotland. Therefore the aim of this study is to address this gap in the literature and investigate the health outcomes and behaviours of the employed, unemployed and the EI populations in Scotland.

## Methods

The 2003 Scottish Health Survey (SHeS) is a cross-sectional national population-based survey and was the latest version available at the time of this study. The survey used a multi-stage stratified probability sampling design, with postcode sectors selected at the first stage and household addresses at the second stage. The survey methodology is described in detail elsewhere [35]. The interview covers a wide range of health items including self-assessed health and disability, cardiovascular and respiratory disease, smoking, drinking, common mental health problems, health service usage, eating patterns and physical activity. The response rate for the interview component of the survey was 60%. This study used the 2003 SHeS to examine the health and health behaviours of the WAP by employment status.

### Employment status

The SHeS asks four questions which are used to derive participant employment status (a. whether working in last week, b. did they do any paid work in the last seven days, c. whether they were looking for paid work in the last four weeks, and d. whether they would have taken paid work in the last two weeks). These questions allow the WAP to be divided into the employed, unemployed and EI and are consistent with the definitions of employment status described in the introduction. We then further subdivided the EI group into three groups – the permanently sick, LAHF and the remaining EI which we have called ‘others’. The early retired were a very small proportion of the sample (2.9%) and were excluded from our study since definite conclusions about whether early retirement is good or bad for an individual’s health are still unclear [7].

### Variables

The selection of SHeS variables used in this study focused on including both objective and subjective measures of health. All variables used in this study are described in Table 1.

**Table 1:** Description of variables used in this study

Variable	Levels	Description
**Health outcomes**		
Self-reported health (SRH)	Good health - 0	**·*** General health* rated as very good, good, fair, bad or very bad
	Poor health −1	**·*** Good health* recoded as very good or good
		**·*** Poor health* recoded as fair, bad, very bad
Limiting longstanding illness (LLI)	No LLI - 0	Respondents asked if they had a limiting-longstanding illness
	LLI - 1	**·*** No LLI*
		**·*** Have LLI*
Mental health	Good mental health - 0	Score from the 12-item General Health Questionnaire (GHQ-12).
	Poor mental health - 1	**·** Score 0–3 recoded as *good mental health*
		**·** Score 4 or more recoded as *poor mental health*
Lung function	Good lung function - 0	Three questions related to respiratory symptoms were asked resulting in the variable ‘wheezed without a cold’.
	Poor lung function - 1	**·** Not ‘wheezed without a cold’ recoded as *good lung function*
		**·** ‘Wheezed without a cold’ recoded *poor lung function*
Smoking status	Non-smoker - 0	Smoking history
	Smoker - 1	**·*** Non-smoker*
		**·*** Current smoker*
Alcohol consumption	Under and including limits - 0	Weekly alcohol consumption (weekly limits: males 21 units, females 14 units)
	Over weekly limits - 1	**·*** Under and including limits*
		**·**`* Over weekly limits*
**Explanatory variable**		
Employment status	Employed −1	Employment status at time of interview
	Unemployed - 2	
	Permanently sick - 3	
	Looking after home and family - 4	
	Others – 5	
**Confounding variables**		
Sex	Male – 1	Sex of the respondent
	Female – 2	
Age	16-24 - 1	Age of respondent
	25–34 - 2	
	35–44 - 3	
	45–54 - 4	
	55–64 - 5	
Deprivation (SIMD quintile)	Scale 1 to 5	The Scottish Index of Multiple Deprivation (SIMD), an area-based level of deprivation, was derived from the residential postcode of the respondents and categorised into quintiles. The SIMD score is calculated at the level of “data zones” using 31 indicators in six individual domains of current income, employment, housing, health, education, skills and training and geographic access to services and telecommunications. Data zones are groups of Census output areas which have populations of between 500 and 1,000 residents.
	Least deprived - 1	
	Most deprived - 5	
Education	Degree or higher −1	Highest level of qualification obtained
	Higher National Certificate/Diploma (HNC/D) or equivalent - 2	
	Higher grade or equivalent - 3	
	Standard grade or equivalent - 4	
	Other school level - 5	
	None - 6	

### Statistical analysis

SPSS version 15 was used for the statistical analyses. The associations between employment status and education and deprivation were analysed using chi-squared tests. Logistic regression was used to assess the dependence of the health outcomes on employment status. The first set of logistic regressions was run unadjusted and the second set was adjusted for age, sex, education and deprivation.

## Results

There were 5877 cases of working age participants (47.2%, n = 2773 females aged 16–59 and 52.8%, n = 3104 males aged 16–64) in the study. The early retired participants (2.9%, n = 172) and missing values (0.4%, n = 25) were excluded leaving 5680 cases. Of these cases the majority of the respondents (71.2%, n = 4045) were in employment. The second largest group were the EI (22.6%, n = 1281), while the unemployed formed the smallest group (6.2%, n = 354). The EI group was further divided into the following three subcategories; permanently sick (35.8%, n = 459), LAHF (41.8%, n = 535) and ‘others’ (22.4%, n = 287). The ‘others’ group consisted of full-time students not looking for paid work, those waiting to take up paid work already obtained, and those intending to look for work but temporarily sick.

The only group which displays a large variation in sex is the LAHF, where 91% of the group are female (Table 2). The mean age of the groups varies from 26.9 for the ‘others’ to 49.1 for the permanently sick (Table 2).

**Table 2:** Sex and age distribution of respondents according to their employment status

**Employment status**	**Male (%)**	**Female (%)**	**Mean age**
Employed	49.7	50.3	41.4
Unemployed	53.1	46.9	31.4
Permanently sick	54.5	45.5	49.1
Looking after home and family	9.0	91.0	40.2
Others	49.1	50.9	26.9

Table 3 shows a statistically significant association between employment status and education and deprivation (as measured by the Scottish Index of Multiple Deprivation, SIMD). There are very large differences in the educational qualifications of the economic groups. 30.4% of the employed have a degree or higher. In comparison only 7.5% of the permanently sick and 13.3% of the LAHF have a degree. There are big variations in the number of respondents having no qualifications (employed 14.6%, permanently sick 51.5%, LAHF 33.9%). 42.7% of the permanently sick group live in the most deprived areas (SIMD 5) compared to 12.9% of the employed, 23.4% of the unemployed and 25.2% of the LAHF. Only 5.4% of the permanently sick live in SIMD 1 (least deprived) in contrast to 22.9% of the employed population.

**Table 3:** Employment status, education and deprivation (percent in each economic group)

Education*	**Degree or higher%**	**HNC/D Or equiv%**	**Higher grade or equiv%**	**Standard grade or equiv%**	**Other school level%**	**No qualifications%**
Employed	30.4	9.9	20.4	19.9	4.8	14.6
Unemployed	14.1	7.3	25.1	31.6	3.1	18.6
Permanently sick	7.5	2.2	14.5	16.1	8.1	51.5
Looking after home and family	13.3	6.0	14.2	25.5	7.1	33.9
Others	13.6	3.1	31.1	31.8	3.8	16.4
Deprivation**	**SIMD 1**^**#**^**%**	**SIMD 2%**	**SIMD 3%**	**SIMD 4%**	**SIMD 5%**	
Employed	22.9	24.9	22.0	17.3	12.9	
Unemployed	13.6	16.1	24.9	22.0	23.4	
Permanently sick	5.4	9.6	16.6	25.7	42.7	
Looking after home and family	15.0	19.1	16.1	24.7	25.2	
Others	19.2	17.1	22.3	18.8	22.6	

* Test of association on education and employment status p-value < 0.001.

** Test of association on deprivation category and employment status p-value < 0.001.

# SIMD 1 is the least deprived and SIMD 5 is the most deprived.

Looking at the adjusted odds ratios (OR) the LAHF group and the ‘others’ are more likely to have poor self-reported health (SRH) than the employed (2.2 times and 2.45 times respectively), but show no difference from the unemployed group (Table 4). However the permanently sick are 25 times more likely to self report poor health than the employed and this perception of health is significantly worse compared to all the other employment groups. The unemployed are 1.7 times more likely to self report poor health than the employed.

**Table 4:** Odds ratios for poor health by employment status

**Variable**	**Unadjusted OR (95% CI)**	Adjusted OR (95% CI)^*^
**Poor self-reported health**		
Employed	1	1
Unemployed	1.54 (1.17-2.02)	1.72 (1.28-2.30)
Permanently sick	40.75 (30.58-54.31)	25.43 (18.90-34.21)
Looking after home and family	2.59 (2.11-3.18)	2.20 (1.76-2.76)
Others	1.80 (1.35-2.40)	2.45 (1.77-3.40)
	p < 0.001	p < 0.001
**Limiting longstanding illness**		
Employed	1	1
Unemployed	1.19 (0.88-1.60)	1.53 (1.11-2.09)
Permanently sick	91.89 (62.44-135.24)	65.59 (44.19-97.33)
Looking after home and family	2.11 (1.71-2.62)	2.03 (1.61-2.56)
Others	1.64 (1.22-2.21)	2.70 (1.92-3.78)
	p < 0.001	p < 0.001
**Poor mental health**		
Employed	1	1
Unemployed	2.04 (1.54-2.71)	2.28 (1.69-3.08)
Permanently sick	7.20 (5.80-8.95)	7.40 (5.80-9.45)
Looking after home and family	1.69 (1.31-2.16)	1.51 (1.16-1.96)
Others	2.65 (1.97-3.56)	3.28 (2.35-4.58)
	p < 0.001	p < 0.001
**Poor lung function**		
Employed	1	1
Unemployed	1.34 (1.02-1.77)	1.34 (1.00-1.80)
Permanently sick	3.49 (2.84-4.29)	3.22 (2.56-4.05)
Looking after home and family	1.14 (0.09-1.45)	1.12 (0.86-1.44)
Others	1.31 (0.96-1.79)	1.41 (1.01-1.97)
	p < 0.001	p < 0.001
**Current smoker**		
Employed	1	1
Unemployed	2.03 (1.62-2.53)	1.67 (1.31-2.12)
Permanently sick	2.85 (2.34-3.46)	1.84 (1.48-2.29)
Looking after home and family	1.91 (1.58-2.29)	1.35 (1.10-1.66)
Others	1.28 (0.99-1.67)	1.11 (0.83-1.49)
	p < 0.001	p < 0.001
**Heavy alcohol consumption**		
Employed	1	1
Unemployed	1.01 (0.78-1.31)	0.84 (0.64-1.11)
Permanently sick	0.70 (0.54-0.89)	0.72 (0.55-0.94)
Looking after home and family	0.40 (0.30-0.53)	0.52 (0.39-0.70)
Others	0.77 (0.56-1.05)	0.61 (0.44-0.86)
	p < 0.001	p < 0.001

*Odds ratio (OR) and 95% confidence interval (CI) adjusted for age, sex, education and deprivation (SIMD).

The LAHF, ‘others’, and unemployed are more likely to have a limiting-longstanding illness than the employed group (2 times and 2.7 times and 1.5 times, respectively), and this difference is even greater for the permanently sick who are 66 times more likely to have a LLI than the employed (Table 4).

With regard to mental health, the LAHF, ‘others’ and unemployed, demonstrate poorer mental health than the employed (1.5 times, 3.3 times and 2.3 times, respectively). The permanently sick are 7.4 times more likely to have poor mental health than the employed. The LAHF have slightly but significantly worse mental health than the employed group and their mental status is significantly different from all other EI groups (LAHF OR 1.51 95% CI 1.16-1.96; permanently sick OR 7.40 95% CI 5.80-9.45; ‘other’ OR 3.28 95% CI 2.35-4.58).

The permanently sick are 3.2 times more likely to have poor lung function compared to the employed, while the ‘others’ are only 1.4 times more likely to report poor lung function compared to the employed. The LAHF were no worse than the employed (Table 4).

The permanently sick, LAHF and unemployed are more likely to be smokers than the employed group (Table 4).

The permanently sick, LAHF, and ‘others’, are less likely to exhibit heavy alcohol consumption compared to the employed. There is no difference in alcohol consumption between the employed and unemployed (Table 4).

## Discussion

This is the first study that has looked at the breakdown of the EI group by a number of health measures in Scotland. This study's main variable of interest is employment status, which distinguishes it from other studies that looked at employment status as a confounding factor and not as the main outcome variable [10,36].

The EI are a significant proportion of the potential workforce and much of the literature have studied them as one single group [22,25,26]. This has resulted in inherent assumptions of homogeneity within this group and therefore important differences between the EI subgroups being undetected and policies not always being appropriate. In this study, the permanently sick made up 36% of the EI population, but the largest group were the LAHF (42%). While the permanently sick did have much worse health than all other economic groups, important results were found for the LAHF. This study highlights that the LAHF, while grouped in with the EI due to their association with the labour market, constitute a rather different group and demonstrate similar traits to the employed and unemployed.

Only more recently has the effect on mental health and psychological wellbeing been investigated in the EI population, however unlike our study they have been treated as one group [26]*.* Our study revealed a relatively better mental status in the LAHF with respect to the remaining EI subgroups which was consistent to a recent English study using the Adult Psychiatric Morbidity Survey 2007 [30]. Similar to our study, Ford et al. (2010) examined mental health status in the EI by breaking down the group [30]. However, their breakdown was based on benefits received [30], and may therefore inadvertently exclude many of the EI, in particular the LAHF who do not receive benefits. The majority of the LAHF group in our study were women. This gender disproportion may provide one explanation for our finding. Women tend to carry out the greater share of domestic responsibilities and therefore, when unemployed or LAHF, their multitasking overload may decrease, which in turn could reduce stress and attenuate the risk of poor mental health [30].

By investigating lung function we were able to include a measure of physical health in addition to perceived health. Our study showed that being in work was not a predictor of good lung function, as the LAHF showed no difference from the employed, whereas the unemployed had slightly worse lung function than those in employment. The results we obtained for the unemployed were comparable to a previous study, where unemployment was associated with increased susceptibility to upper respiratory infections [37].

The permanently sick, LAHF and unemployed are more likely to be smokers than the employed group which is consistent with the findings of a longitudinal study which showed that men who experienced unemployment were more likely to smoke [38]. Also the non employed do not have the workplace smoking restrictions which have been shown to decrease smoking prevalence [39]. In this study all groups within the EI population were less likely to exhibit heavy alcohol consumption compared to the employed. Economic reasons and under-reporting consumption levels [40] may provide possible explanations as it has been estimated that population surveys can underestimate true levels of alcohol consumption by approximately 50% in Scotland [40]. This is in contrast to a previous study in Scotland that found that being unemployed, retired or EI was a significant predictor of alcohol-related hospitalisations compared to those in employment [36]. However alcohol-related hospitalisations are a much later event than measuring current alcohol consumption.

The impact of unemployment on health can be modified by a number of socio-economic factors [7,30]. The role of educational attainment on health and health behaviours is not fully understood. Although potential mechanisms have been proposed [10,41], this study seeks only to describe educational attainment by economic group. The permanently sick showed much poorer educational attainment. The LAHF had a higher proportion with no qualifications than the unemployed, but were more alike in all other educational categories. This lack in educational qualifications in the permanently sick and LAHF, aside from health issues, may be a consequence of socio-economic circumstances or caring responsibilities in early adulthood, which inhibit them from progressing beyond school qualifications.

Although employment, education skills and training are two of the six domains used to derive the SIMD, the comparison of employment status by SIMD reveals interesting findings. The highest proportion of the permanently sick live in the most deprived areas, yet the LAHF are more evenly distributed across the five quintiles but still live in more deprived areas with respect to the employed. This further illustrates the fact that the EI are not a homogeneous group and reinforces the need to examine their characteristics and needs separately. This divide in the distribution of the EI subgroups by SIMD category can partly be explained by the fact that many of the LAHF are in this group by choice, may be financially secure and not dictated there due to ill health.

Separating the EI group has shown important differences in health. A review study has shown that ‘employment’ is overall good for health and wellbeing [7]. However, the available evidence is mainly about paid employment. But work is much more than this. Work includes unpaid and voluntary work, education and training, family responsibilities and caring [7,30]. The fact that in this study the LAHF show better health outcomes than the permanently sick but roughly similar to the unemployed demonstrates the benefits of engaging in purposeful activity.

There are limitations of the data used in this study particularly with the LAHF group. The LAHF are predominantly women and although we have discussed the considerable heterogeneity of this group this study only looks at individual status and is not able to consider their marital/partnership status, if they live in a household where their partner’s income is sufficient enough to make their involvement in the labour market unnecessary or if they are lone parents unable to work because of unaffordable childcare costs. Another limitation is that we used data from 2003 which predates the current recession. Evidence suggests psychological ill health, LLI and poor SRH all appear to increase during economic recessions but there is also the suggestion that health behaviours may improve especially tobacco and alcohol consumption [11]. The size of the EI group is therefore more likely to be larger and the more recent Scottish Health Surveys (the survey became continuous in 2008 and now report annually) could now be used to investigate health effects of the recession.

In the past most studies have focussed on the employed and the unemployed. The employed are often the target of health promotion, health protection and vocational rehabilitation efforts [42] and the unemployed also have compulsory employability and skills training in order to receive benefits. Previously the EI were often a largely ignored part of the labour market. However, there have been major changes in the welfare system in the UK and Scotland and some attempts are being made to assist these groups. Changes in government policy have seen the introduction of measures aimed at helping the permanently sick back into the labour market (e.g. New Deal for Disabled People, Pathways to Work). Further, with the introduction of ESA in 2008, many of the permanently sick now have to engage with employability and rehabilitation services [33,34], though this reform of the sickness benefit system signifies a dangerous political shift in how ill and disabled patients are seen as either ‘deserving’ or ‘undeserving’ of state support [11]. Similarly, a substantial proportion of EI lone parents are also being targeted by policy reform [32]. Also many of the LAHF group who do not claim benefits will not be required to engage with these new employability programmes and while many may not want to work, improving the health and education/skills of the LAHF group does needs consideration, as this group have an important role of educating and maintaining the health of their own children.

The findings of this study have important policy implications for the health strategies focused on the WAP and in particular the EI. Similar to other studies [27-30] our findings further indicate that the EI group is at higher risk for ill health. They are part of the potential workforce and should not be ignored in public health policies aimed at improving the health of the WAP. Returning to work has proved beneficial for recovery from LLI and poor mental health regardless of socio-economic circumstances [22,29] and therefore national vocational rehabilitation strategies could help a proportion of the EI become work ready, improve their health and alleviate health inequalities. Even though there are programmes in place in the current policy framework [33,34], there are still people within the EI group who could benefit from better tailored services and available research evidence suggests that a ‘health first’ approach to welfare reform is potentially the most effective [11]. A better health status of the entire WAP would benefit both the individual and society.

## Conclusion

The results from this study contribute to the evidence that health varies by employment status. Importantly we have been able to address the different groups within the workless population and indicate associations of varying strengths between health variables and employment status. This study suggests that the EI group is at higher risk and policies and strategies directed at this group may need particular attention.

## Abbreviations

EI: Economically inactive; ESA: Employment and support allowance; HNC/D: Higher national certificate/diploma; ILO: International labour organisation; LAHF: Looking after home and family; LLI: Limiting longstanding illness; OR: Odds ratio; SHeS: Scottish health survey; SIMD: Scottish index of multiple deprivation; SRH: Self-reported health; WAP: Working age population.

## Competing interests

The authors declare they have no competing interests.

## Authors’ contributions

JB designed the study and coordinated the work. MAT carried out the initial literature review, undertook the analyses and interpreted the results. MAT and HG undertook the statistical analyses. ED & JB were the main authors of the manuscript with all authors contributing and commenting on the final version. All authors read and approved the final manuscript.

## Pre-publication history

The pre-publication history for this paper can be accessed here:

http://www.biomedcentral.com/1471-2458/12/327/prepub
